# Exosome surface glycans reflect osteogenic differentiation of mesenchymal stem cells: Profiling by an evanescent field fluorescence-assisted lectin array system

**DOI:** 10.1038/s41598-019-47760-x

**Published:** 2019-08-08

**Authors:** Asako Shimoda, Shin-ichi Sawada, Yoshihiro Sasaki, Kazunari Akiyoshi

**Affiliations:** Department of Polymer Chemistry, Graduate School of Engineering, Kyoto University, Katsura, Nishikyo-ku, Kyoto, 615-8510 Japan

**Keywords:** Glycobiology, Mesenchymal stem cells

## Abstract

Extracellular vesicles (EVs) carry information between cells in the form of biomolecules. Such molecules have been found to serve as biomarkers. Glycans attached to surface molecules on EVs are involved in their cellular uptake. In this study, we examined glycan profiles of small EVs which are generally termed exosomes before and after osteogenic differentiation of adipose-derived mesenchymal stem cells (MSCs) by an evanescent field fluorescence-assisted (EFF)-lectin array system to discover glycan biomarkers for osteogenic differentiation. We found few differences between exosomes before and after osteogenic differentiation of MSCs in terms of fundamental characteristics such as size, morphology, and exosomal marker proteins. However, specific lectins bound strongly to exosomes from differentiated cells. Exosomes from osteogenically differentiated MSCs bound strongly to fucose- and mannose-binding lectins, especially at a high concentration of exosomes. In summary, we found that several lectins bound to exosomes from differentiated MSCs more strongly than to those from undifferentiated cells using an EFF-lectin array system, indicating that monitoring exosomal surface glycans may identify predictive indexes of osteogenic differentiation.

## Introduction

“Extracellular vesicles (EVs)” is the generic name of cell-derived membrane vesicles including exosomes, microvesicles, oncosomes, and apoptotic bodies^1^. Exosomes, a type of small extracellular vesicles which are recovered by ≥100,000 g ultracentrifugation, are formed in the process of the endocytosis pathway^1^. All types of cells release exosomes that carry biological information in their components, especially proteins and microRNAs (miRNAs), to other cells. Based on this important function for cell-to-cell communication, exosomes are thought to be fingerprints of their originating cells, which can be used as biomarkers for diagnosis, prognosis, and determining the cell state^2^. For example, exosomal circulating miRNAs have been found in various kinds of body fluids, including serum, plasma, and urine, especially in cancer patients. Tumour-related miRNAs are found in serum exosomes of glioblastoma^3^ and non-small cell lung cancer patients^4^, in plasma exosomes of breast cancer patients^5^ and colon cancer patients^6^, and in urine exosomes of prostate cancer patients^7^. Similarly, exosomal proteins are also considered as diagnostic biomarkers of cancer^8^ and infection^9^, and lipids in urine exosomes are being reported as emerging markers of prostate cancer^10,11^.

The cell membrane is densely coated with glycans that are normally attached to proteins and lipids to form glycoproteins and glycolipids, respectively. Cell surface glycans participate in a wide variety of biological functions such as cell-cell interactions, protein folding, immune regulation, and virus infection^12^. Recent studies have shown that exosomes are typically internalized into recipient cells through multiple endocytic routes or membrane fusion. Cell-exosome interactions are mediated by various proteins including transmembrane proteins, extracellular matrix proteins, and proteoglycans^13^. These findings suggest that glycans attached to surface molecules on exosomes are involved in cellular uptake of exosomes. We found that exosomes from human adipose-derived mesenchymal stem cells (MSCs) strongly interact with sialic acid-binding lectins, and sialic acids on exosomes are involved in cellular uptake of exosomes *in vitro* and *in vivo*^14^.

To elucidate further functions of exosomal surface glycans in biological events, we examined glycan profiles resulting from MSC differentiation, particularly during osteogenic differentiation. It is well known that MSCs can differentiate into osteoblasts, adipocytes, chondrocytes, neurons, and myocytes, of which the first three cell types have been particularly well studied^15^. Bone remodelling is the process through which bones are continuously regenerated by maintaining the balance of bone resorption and formation to maintain homeostasis^16^. Unbalanced bone remodelling can cause various bone disorders including osteoporosis, Paget disease, and heterotopic ossification^16,17^. Therefore, understanding the bias in the bone remodelling balance is important to avoid the risk of these diseases. Osteoblasts are responsible for bone formation, and some protein markers, such as alkaline phosphatase (ALP), osteocalcin, and type I collagen, are thought to be useful as bone formation biomarkers^18^. However, using these markers has several practical problems: (1) ALP and type I collagen are not specific to bone; (2) Expression may change in response to environmental factors, including the time of day, season, food, diseases, and drugs^19,20^. To overcome these limitations, a new biomarker is needed for bone formation. As mentioned above, exosomal components can be various kinds of biomarkers. However, a limited number of studies have reported the role of exosomal glycans to monitor the cell state^21–23^.

Analysis of glycan patterns on EVs from various types of cells was reported by Batista *et al*. in 2011^24^. They showed that cell-specific and EV-enriched glycan patterns on EVs from T-cells, melanoma, colon cancer, and breast milk. Additionally, Liang *et al*. found that glycosylation is important for glycoprotein sorting into EVs^25^. Our previous study first showed that comprehensive glycan patterns on intact exosomes can be analysed using an evanescent field fluorescence-assisted (EFF) lectin array system^14^. The advantages of this method especially by using EFF lectin array system are as follows: (1) Glass slides spotted with dozens of lectins (an array with 45 lectins was used for this study) enable determination of glycan patterns simultaneously; (2) Washing steps to remove unbound samples are unnecessary because the area of the evanescent field is confined to the immediate vicinity of the glass (<150 nm); (3) Rapid and simple processing of a small amount of sample are superior to other processes such as those in mass spectrometry, high performance liquid chromatography, nuclear magnetic resonance, and capillary electrophoresis^26^.

Here, we hypothesized that specific glycan biomarkers for osteogenic differentiation of adipose-derived MSCs can be detected by profiling of exosome surface glycans by an EFF-lectin array system. Undifferentiated MSC- and osteogenically differentiated MSC-derived exosomes were collected and their surface glycans were analysed using an EFF-lectin array method. We found that several lectins bound to exosomes from differentiated cells more strongly than to those from undifferentiated cells, indicating that monitoring exosomal glycans using an EFF-lectin array may discriminate not only cellular differentiation, but also reprogramming, pluripotency, and the cancer stage.

## Results

### Isolation and characterisation of MSC- and osteogenically differentiated MSC-derived exosomes

**Figure 1:**
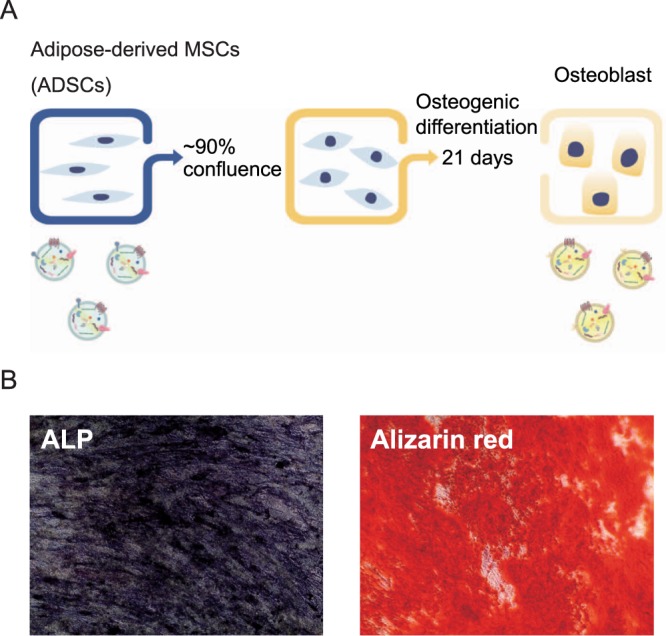
(**A**) Scheme of osteogenic differentiation of MSCs. (**B**) ALP and Alizarin red staining of osteogenically differentiated MSCs on day 21.

Differentiation from MSCs to osteoblasts was induced by culture in growth medium supplemented with dexamethasone, (+)-sodium L-ascorbate, and β-glycerophosphate disodium. ALP and Alizarin red staining after 21 days of osteogenic induction revealed that cells were successfully differentiated into mineralized osteoblasts (Fig. 1).

**Figure 2:**
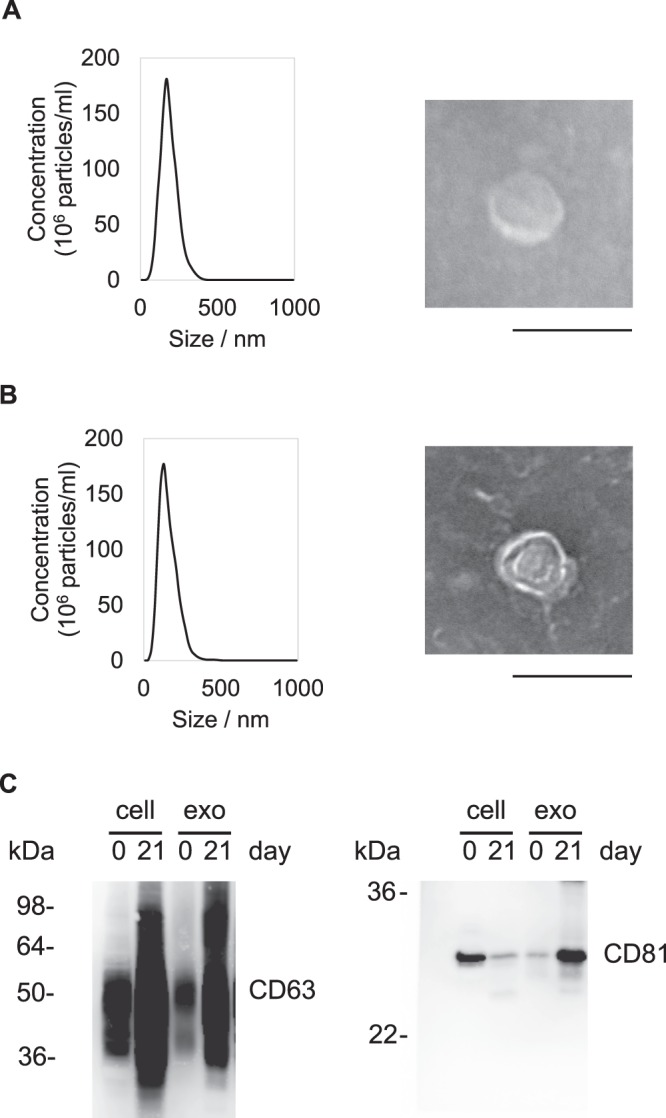
Characterization of exosomes from MSCs (**A**) and osteogenically differentiated MSCs (**B**). The size distribution and morphology of exosomes were determined by NTA and TEM, respectively. Data represent the mean ± standard deviation (SD) of three (MSCs) or five (osteogenically differentiated MSCs) independent experiments. Scale bar = 100 nm. (**C**) Exosome markers (CD63 and CD81) were detected in cell lysates and exosomes after osteogenic differentiation induction at days 0 and 21. Uncropped blot images are shown in Supplementary Fig. S1.

Exosomes from undifferentiated or osteogenically differentiated MSCs were isolated by ultracentrifugation and analysed for their size distributions, morphologies, and exosome markers (CD63 and CD81). Lipid bilayer vesicles were observed by transmission electron microscopy (TEM), and nanoparticle tracking analysis (NTA) revealed that the average diameters of MSC exosomes and osteogenically differentiated MSC exosomes were 181 ± 11 nm and 156 ± 12 nm, respectively (Fig. 2A,B). In addition, the number of particles at 1 µg/mL obtained by NTA was 6.5 ± 3.0 × 10^8^ particles/mL (MSC exosomes) and 7.7 ± 2.4 × 10^8^ particles/mL (osteogenically differentiated MSC exosomes). CD63 and CD81 in exosomes and cell lysates was confirmed by western blot analyses (Fig. 2C, full-length blots are presented in Supplementary Fig. S1).

### Surface glycan patterns before and after osteogenic differentiation determined by an EFF-lectin array

**Figure 3:**
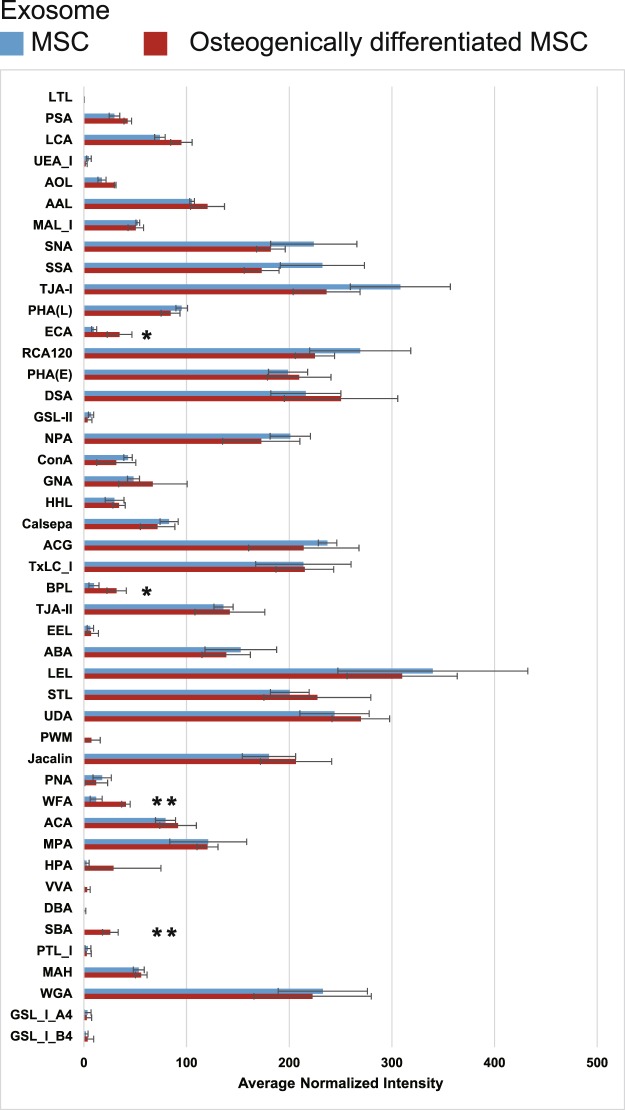
Glycan profiles of exosomes from undifferentiated and osteogenically differentiated MSCs on day 21. Each fluorescence intensity was normalized to the average intensities of all lectins. Data represent the mean ± SD of three independent experiments, *p < 0.05, **p < 0.01.

**Figure 4:**
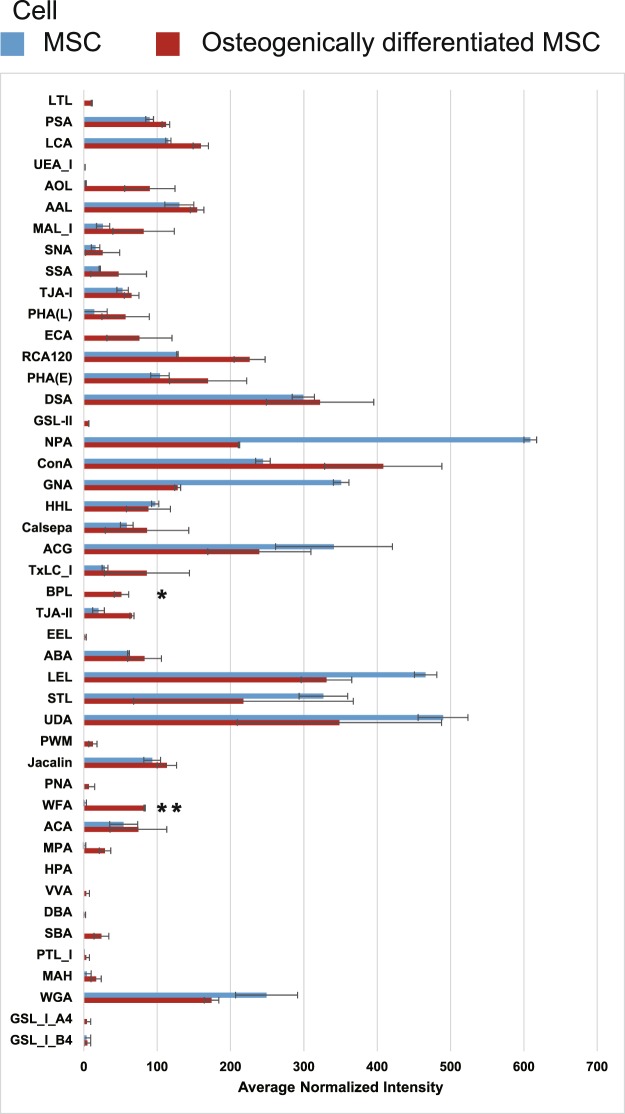
Glycan profiles of undifferentiated and osteogenically differentiated MSCs on day 21. Each fluorescence intensity was normalized to the average intensities of all lectins. Data represent the mean ± SD of two independent experiments, *p < 0.05, **p < 0.01.

Cy3 dye-labelled plasma membrane proteins from MSCs or osteogenically differentiated MSCs and intact exosomes from both cell types were prepared to analyse glycan patterns using the EFF-lectin array. The samples were added to each well on a lectin array containing 45 lectins (Supplementary Table S1), and each fluorescence intensity was normalized to the average intensities of the 45 lectins. As we found in our previous study, exosomes were more strongly bound to α2-6 sialic-acid-recognizing lectins [*Sambucus nigra* (SNA), *Sambucus sieboldiana* (SSA), and *Trichosanthes japonica* (TJA-I)], which was common to both undifferentiated and differentiated MSCs (Supplementary Figs S2 and 3). Next, we evaluated the difference between exosomes from MSCs and osteogenically differentiated MSCs. Four lectins (ECA (Galβ1-4GlcNAc), BPL (terminal *β*-GalNAc), WFA (terminal *β*-GalNAc), and SBA (terminal *β*-GalNAc)) had statistically higher binding affinities (>3-fold change, *P < 0.05 and **P < 0.01) to exosomes from osteogenically differentiated MSCs than to those from undifferentiated MSCs (Fig. 3). In cell membrane fractions, increasing BPL and WFA binding affinities were observed during osteogenic differentiation (>3-fold change, *P < 0.05 and **P < 0.01, Fig. 4).

**Figure 5:**
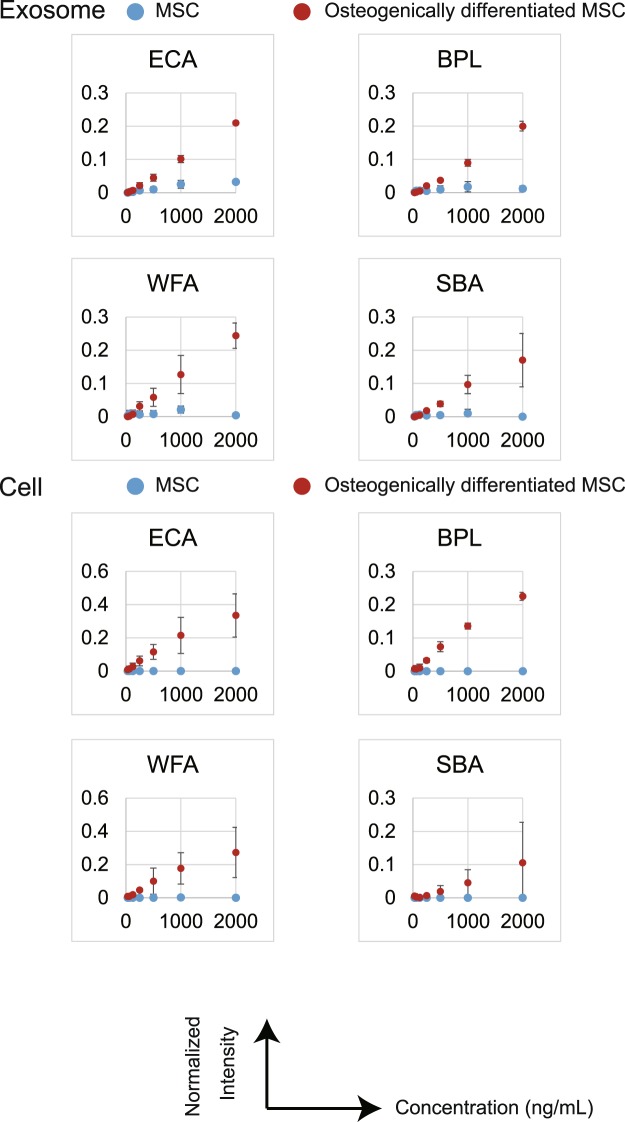
Binding curves of interactions between four lectins (ECA, BPL, WFA, and SBA) and exosomes or cell membrane fractions. The resulting signals were normalized to the signal of LEL lectin. Data represent the mean ± SD of two independent experiments.

**Figure 6:**
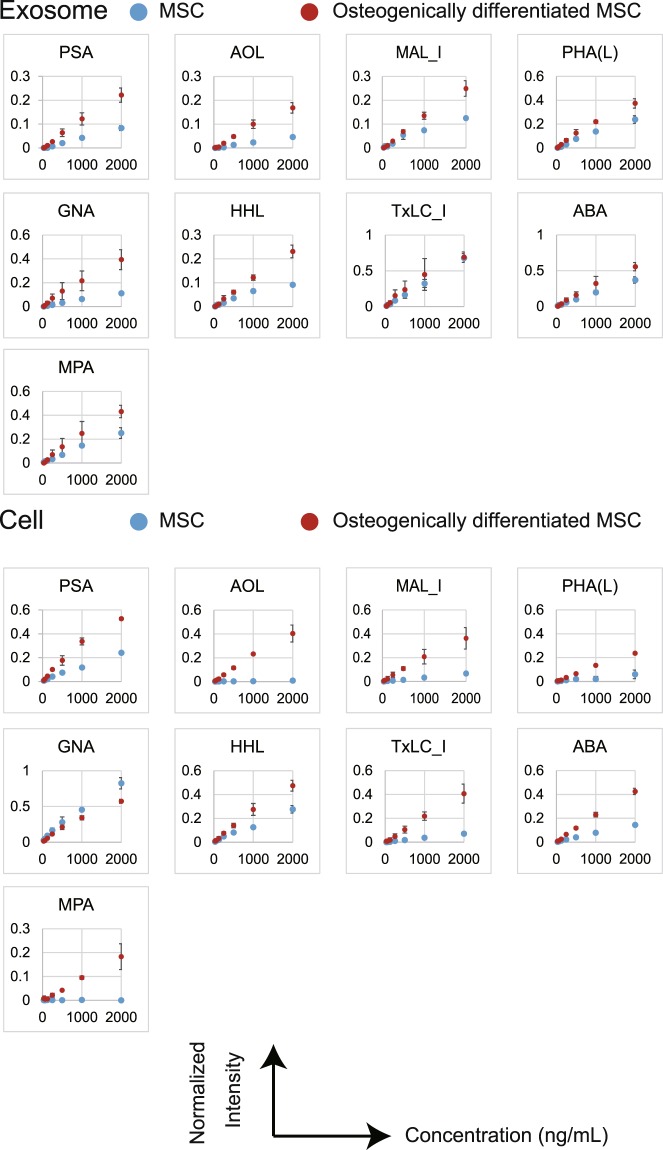
Binding curves of interactions between nine lectins (PSA, AOL, MAL_I, PHA(L), GNA, HHL, TxLC_I, ABA and MPA) and exosomes or cell membrane fractions. The resulting signals were normalized to the signal of LEL lectin. Data represent the mean ± SD of two independent experiments.

To obtain further insights into the affinity for lectins, we investigated the binding curves for each of the 45 lectins with cells or exosomes as a function of the concentrations from undifferentiated and osteogenically differentiated MSCs (Supplementary Figs S4 and 5). The resulting signals were normalized to the signal of one specific lectin, *Lycipersicon esculentum* (LEL), whose signal did not change during differentiation, as a reference^27^. Similarly to the results shown in Figs 3 and 4, four lectins (ECA, BPL, WFA, and SBA) strongly bound to osteogenically differentiated MSC-derived exosomes and cells with the increase in sample concentration (Fig. 5). Furthermore, other four lectins (PSA, AOL, GNA and HHL) showed higher binding to exosomes from osteogenically differentiated MSCs, and six lectins (AOL, MAL_I, PHA(L), TxLC_I, ABA, and MPA) showed higher binding to cells from osteogenically differentiated MSCs in accordance with the increase in sample concentration (Fig. 6).

## Discussion

Because the characteristics of exosomes are known to reflect their cell of origin, they are attracting attention as biomarkers for diagnosis and treatment of diseases (especially cancer) and to determine the cell state. Most studies have concentrated on exosomal miRNAs or proteins, and there is little information about the role of exosomal glycans, mainly because glycans have much more complex structures than those of other biomolecules (e.g. DNAs, miRNAs, and proteins). Furthermore, specialized equipment is required, as well as complicated sample pretreatment, multiple samples, and long analysis time for their structure determination^28^. In our previous study, we found that an EFF-lectin array method is excellent for analysis of exosomal glycans in terms of both operability and sensitivity compared with typical approaches such as MS and HPLC. As another advantage, glycan-lectin interactions can be simply detected by adding intact fluorescence-modified exosomes to the array without any special processing.

Because exosomes, especially those derived from MSCs, are considered to be novel candidates for cell-free therapy^29^, we further showed that the surface glycans on exosomes play important roles in cellular uptake and distribution^14^. To discover additional functions of exosomal glycans, in this study, we examined differences in surface glycan patterns on exosomes before and after induced differentiation of MSCs. Some glycoproteins and glycolipids are known as stem cell markers^30^, and specific changes in surface glycans of stem cells have been reported. In mouse embryonic stem cells, the lectin binding profile is considered to be an ideal indicator of differentiation^31–33^. In MSCs, α2-6-sialylated N-glycans have been reported to be an index indicating whether MSCs have differentiation potentials^34^. Furthermore, it has been shown that glycan patterns of osteogenically and adipogenically differentiated MSCs are different from those of undifferentiated MSCs^35,36^. The number of reports on the roles of exosomes in bone remodeling have been increasing in recent years, which mainly focus on the differences in exosomal protein^37–39^ and miRNA^40,41^ profiles between pre-osteoblasts and mineralizing osteoblasts, or evaluate cellular interactions with them. Because growth factors released by osteoblasts promote the proliferation of prostate cancer cells^42^, Morhayim *et al*. found that osteoblast-derived exosomes are efficiently internalized into prostate cancer cells^38^, and Bilen *et al*. revealed involvement of cadherin-11 in cellular uptake of exosomes^39^. Furthermore, receptor activator of nuclear factor κB ligand (RANKL), which is expressed in osteoblasts, was identified in osteoblast-derived exosomes. RANKL-positive exosomes were shown to be taken up by osteoclasts, resulting in the induction of osteoclastic differentiation from monocytes^43,44^. These findings suggest that osteoblasts deliver their biological information through exosomes to target cells.

In the current study, we substituted exosomes for plasma membranes to identify novel biomarkers of osteogenic differentiation of MSCs. There were not many differences between exosomes before and after osteogenic differentiation of MSCs in terms of fundamental characteristics (size, morphology, and exosomal marker proteins), while specific lectins strongly bound to exosomes from differentiated cells. An EFF-lectin array method assists comprehensive analysis of multiple samples simultaneously and estimates the difference between them. We also evaluated the binding affinities of cell membrane fractions and exosomes using binding curves. This evaluation yielded the same results that both cell membrane fractions and exosomes from osteogenically differentiated MSCs displayed high affinity for four lectins (ECA, BPL, WFA, and SBA). Interestingly, higher affinities interactions were observed between some lectins and exosomes or cell membrane fractions, especially at a high concentration of samples, indicating that analysis of exosome surface glycans by the EFF-lectin array system may identify predictive indexes of osteogenic differentiation.

Few reports have focused on the change in the glycan pattern during biological events. Gerlach *et al*. examined glycan profiles of urinary extracellular vesicles (uEVs) by a lectin microarray and found that surface glycans on uEVs can be used as biomarkers of polycystic kidney disease^22^. Moyano *et al*. analysed glycolipids (sulfatides) in plasma exosomes from multiple sclerosis patients and found that C16:0 sulfatide levels were higher than in healthy samples^45^. There are some studies of the changes in cell surface glycans during osteogenic differentiation. Xu *et al*. proposed that α2-3 sialic-acid expression on the pre-osteoblast cell surface was important for osteoblast mineralization^46^. Wilson *et al*. analyzed *N*-glycan profiles of MSCs and osteoblasts by mass spectrometry and found that oligomannose and fucose on antennae structures on MSCs are important to maintain their stem cell potential^47^. Because the role of exosomal glycans during cell differentiation has not been clarified so far, our data are the first to indicate their possible application as a novel cell differentiation index. Although we only revealed the differences in glycan profiles of undifferentiated and osteogenically differentiated MSCs, the methods are useful for application to other cells types such as various kinds of cancer cells.

## Materials and Methods

### Cell cultures

Adipose-derived MSCs were obtained from Lonza (Walkersville, MD, USA) and cultured in serum free StemPro® MSC SFM XenoFree medium (Thermo Fisher Scientific, Waltham, MA, USA) containing CTS™ GlutaMAX™-I (Thermo Fisher Scientific) at 37 °C in an atmosphere with 5% CO_2_. For osteogenic differentiation, passage 3–6 ADSCs were cultured to 90% confluence, and then the culture medium was replaced with osteogenic medium [MSC growth medium supplemented with 5 nM dexamethasone (Sigma-Aldrich, St. Louis, MO, USA), 250 μM (+)-sodium L-ascorbate (Sigma-Aldrich), and 10 mM β-glycerophosphate disodium (Sigma-Aldrich)]. The medium was changed two or three times a week, and the cells were cultured for 21 days.

### ALP and alizarin red S staining

To evaluate osteogenic differentiation, MSCs were seeded at a density of 5000 cells/cm^2^ in a 24-well cell culture plate and cultured until 90% confluence. After 21 days of osteogenic induction, ALP and Alizarin Red S staining were performed using a TRACP & ALP double-stain Kit (TAKARA BIO Inc., Shiga, Japan) and Calcification Evaluation Set (Iwai Chemicals Co., Ltd., Tokyo, Japan), according to the manufacturers’ instructions, respectively.

### Isolation of MSC- and osteogenically differentiated MSC-derived exosomes

For MSC-derived exosome isolation, subconfluent cells were cultured in fresh growth medium for 48 h before collecting the supernatant. For osteogenically differentiated MSC-derived exosomes, the medium was replaced with fresh medium at day 19 after osteogenic induction, and then the cells were incubated for a further 48 h. The resulting conditioned media from both cell types were centrifuged at 300 × *g* for 10 min, 2,000 × *g* for 10 min, 10,000 × *g* for 30 min, and then 120,000 × *g* for 100 min at 4 °C. Subsequently, the pellets were washed with phosphate-buffered saline (PBS) by ultracentrifugation at 120,000 × *g* for 100 min at 4 °C. Exosome pellets were resupended in PBS and stored at −80 °C until use. The concentration of exosomal proteins was determined using a Micro BCA assay kit (Pierce, Rockford, IL, USA).

### Nanoparticle tracking analysis (NTA)

The size distribution of exosomes was determined by NTA. The exosome solution was diluted to a concentration of 4–8 × 10^8^ particles/mL and analysed using a NanoSight LM10 (Malvern Instruments Ltd, UK) with a blue laser. Experimental conditions were as follows: Measurement Time: 60 s; Blur: Auto; Detection Threshold: 4–5; Min Track Length: Auto; Min Expected Size: Auto. Data represent the mean ± standard deviation (SD) of three (MSCs) or five (osteogenically differentiated MSCs) independent experiments.

### Western blotting

Cell lysates in RIPA buffer (Nacalai Tesque Inc., Kyoto, Japan) and exosomes were separated by 12.5% SDS-PAGE under non-reducing conditions. The separated proteins were transferred onto polyvinylidene difluoride membranes with an iBlot 2 Dry Blotting System (Thermo Fisher Scientific). After blocking with Blocking-One (Nacalai Tesque Inc.) for 30 min, membranes were incubated with the following primary antibodies: anti-CD63 (ab59479; Abcam, Cambridge, UK) and anti-CD81 (Thermo Fisher Scientific). After overnight incubation at 4 °C, the membranes were incubated with horseradish peroxidase-conjugated secondary antibodies and EzWestLumi plus (ATTO, Tokyo, Japan). Proteins bands were visualized using a LAS-4000 (GE Healthcare).

### Transmission electron microscopy (TEM)

The morphologies of MSC- and osteogenically differentiated MSC-derived exosomes were observed using an HT7700-TEM (Hitachi, Tokyo, Japan), as described previously^13^.

### Lectin array

Plasma membrane proteins from MSCs and osteogenically differentiated MSCs were prepared using a Mem-PER™ Plus Membrane Protein Extraction Kit (Thermo Fisher Scientific). Cells and exosomes were labelled with a Cy3 Mono-Reactive dye pack (GE Healthcare Ltd., Tokyo, Japan). Samples were diluted with Probing Solution containing 0.005% Triton X-100 (GlycoTechnica, Yokohama, Japan) to 500–1000 ng/mL and then applied to each well of a lectin microarray chip (LecChip™; GlycoTechnica). After overnight incubation at room temperature, fluorescence images were obtained using a GlycoStation™ Reader 2200 (GlycoTechnica). Data were analysed using GlycoStation® Tools Pro Suite 1.5 (GlycoTechnica).

### Statistical analysis

Statistical analysis was performed using the Student’s t-test. P-values of less than 0.05 were considered as significant.

## Supplementary information

Supplementary Information

## Data Availability

All data generated or analysed during this study are included in this published article and its supplementary information file.

## References

[1] Théry C (2018). Minimal information for studies of extracellular vesicles 2018 (MISEV2018): a position statement of the International Society for Extracellular Vesicles and update of the MISEV2014 guidelines. J Extracell Vesicles..

[2] Colombo M, Raposo G, Théry C (2014). Biogenesis, secretion, and intercellular interactions of exosomes and other extracellular vesicles. Annu. Rev. Cell Dev. Biol..

[3] Skog J (2008). Glioblastoma microvesicles transport RNA and proteins that promote tumour growth and provide diagnostic biomarkers. Nat. Cell Biol..

[4] Grimolizzi F (2017). Exosomal miR-126 as a circulating biomarker in non-small-cell lung cancer regulating cancer progression. Sci. Rep..

[5] Hannafon BN (2016). Plasma exosome microRNAs are indicative of breast cancer. Breast Cancer Res..

[6] Wang J (2017). Circulating exosomal miR-125a-3p as a novel biomarker for early-stage colon cancer. Sci. Rep..

[7] Minciacchi VR, Zijlstra A, Rubin MA, Di Vizio D (2017). Extracellular vesicles for liquid biopsy in prostate cancer: where are we and where are we headed?. Prostate Cancer Prostatic Dis..

[8] Li A, Zhang T, Zheng M, Liu Y, Chen Z (2017). Exosomal proteins as potential markers of tumor diagnosis. J. Hematol. Oncol..

[9] Zhang W (2018). Exosomes in Pathogen Infections: A Bridge to Deliver Molecules and Link Functions. Front Immunol..

[10] Skotland T (2017). Molecular lipid species in urinary exosomes as potential prostate cancer biomarkers. Eur. J. Cancer..

[11] Yang JS, Lee JC, Byeon SK, Rha KH, Moon MH (2017). Size Dependent Lipidomic Analysis of Urinary Exosomes from Patients with Prostate Cancer by Flow Field-Flow Fractionation and Nanoflow Liquid Chromatography-Tandem Mass Spectrometry. Anal. Chem..

[12] Varki A (2017). Biological roles of glycans. Glycobiology..

[13] French KC, Antonyak MA, Cerione RA (2017). Extracellular vesicle docking at the cellular port: Extracellular vesicle binding and uptake. Semin. Cell Dev. Biol..

[14] Shimoda A, Tahara Y, Sawada S, Sasaki Y, Akiyoshi K (2017). Glycan profiling analysis using evanescent-field fluorescence-assisted lectin array: Importance of sugar recognition for cellular uptake of exosomes from mesenchymal stem cells. Biochem. Biophys. Res. Commun..

[15] Chen Q (2016). Fate decision of mesenchymal stem cells: adipocytes or osteoblasts?. Cell Death Differ..

[16] Feng X, McDonald JM (2011). Disorders of bone remodeling. Annu Rev Pathol..

[17] Raisz LG (1999). Physiology and pathophysiology of bone remodeling. Clin. Chem..

[18] Seibel MJ (2005). Biochemical markers of bone turnover: part I: biochemistry and variability. Clin, Biochem, Rev..

[19] Shetty S, Kapoor N, Bondu JD, Thomas N, Paul TV (2016). Bone turnover markers: Emerging tool in the management of osteoporosis. Indian J. Endocrinol. Metab..

[20] Vervloet MG (2017). Circulating markers of bone turnover. J. Nephrol..

[21] Gerlach JQ (2013). Surface glycosylation profiles of urine extracellular vesicles. PLoS One..

[22] Gomes J (2015). Extracellular Vesicles from Ovarian Carcinoma Cells Display Specific Glycosignatures. Biomolecules..

[23] Saito S, Hiemori K, Kiyoi K, Tateno H (2018). Glycome analysis of extracellular vesicles derived from human induced pluripotent stem cells using lectin microarray. Sci. Rep..

[24] Batista BS, Eng WS, Pilobello KT, Hendricks-Muñoz KD, Mahal LK (2011). Identification of a conserved glycan signature for microvesicles. J Proteome Res..

[25] Liang Y (2014). Complex N-Linked Glycans Serve as a Determinant for Exosome/Microvesicle Cargo Recruitment. J Biol Chem..

[26] Zhang L, Luo S, Zhang B (2016). Glycan analysis of therapeutic glycoproteins. MAbs..

[27] Tateno H, Kuno A, Itakura Y, Hirabayashi J (2010). A versatile technology for cellular glycomics using lectin microarray. Methods Enzymol..

[28] Zhang P (2016). Challenges of glycosylation analysis and control: an integrated approach to producing optimal and consistent therapeutic drugs. Drug Discov. Today..

[29] Phinney DG, Pittenger MF (2017). Concise Review: MSC-Derived Exosomes for Cell-Free Therapy. Stem Cells..

[30] Karsten U, Goletz S (2014). What makes cancer stem cell markers different?. Springerplus..

[31] Wearne KA, Winter HC, O’Shea K, Goldstein IJ (2006). Use of lectins for probing differentiated human embryonic stem cells for carbohydrates. Glycobiology..

[32] Nash R, Neves L, Faast R, Pierce M, Dalton S (2007). The lectin Dolichos biflorus agglutinin recognizes glycan epitopes on the surface of murine embryonic stem cells: a new tool for characterizing pluripotent cells and early differentiation. Stem Cells..

[33] Dodla MC (2011). Differing lectin binding profiles among human embryonic stem cells and derivatives aid in the isolation of neural progenitor cells. PLoS One..

[34] Hasehira K, Hirabayashi J, Tateno H (2017). Structural and quantitative evidence of α2-6-sialylated N-glycans as markers of the differentiation potential of human mesenchymal stem cells. Glycoconj. J..

[35] Heiskanen A (2009). Glycomics of bone marrow-derived mesenchymal stem cells can be used to evaluate their cellular differentiation stage. Glycoconj. J..

[36] Hamouda H (2013). N-Glycosylation Profile of Undifferentiated and Adipogenically Differentiated Human Bone Marrow Mesenchymal Stem Cells: Towards a Next Generation of Stem Cell Markers. Stem Cells Dev..

[37] Ge M, Ke R, Cai T, Yang J, Mu X (2015). Identification and proteomic analysis of osteoblast-derived exosomes. Biochem. Biophys. Res. Commun..

[38] Morhayim J (2015). Proteomic signatures of extracellular vesicles secreted by nonmineralizing and mineralizing human osteoblasts and stimulation of tumor cell growth. FASEB J..

[39] Bilen MA (2017). Proteomics Profiling of Exosomes from Primary Mouse Osteoblasts under Proliferation versus Mineralization Conditions and Characterization of Their Uptake into Prostate Cancer Cells. J. Proteome Res..

[40] Morhayim J (2017). Molecular characterization of human osteoblast-derived extracellular vesicle mRNA using next-generation sequencing. Biochim. Biophys. Acta Mol. Cell Res..

[41] Wang X (2018). Mesenchymal stem cell-derived exosomes have altered microRNA profiles and induce osteogenic differentiation depending on the stage of differentiation. PLoS One..

[42] Logothetis CJ, Lin SH (2005). Osteoblasts in prostate cancer metastasis to bone. Nat Rev Cancer..

[43] Deng L (2015). Osteoblast-derived microvesicles: A novel mechanism for communication between osteoblasts and osteoclast. Bone..

[44] Cappariello A (2018). Osteoblast-Derived Extracellular Vesicles Are Biological Tools for the Delivery of Active Molecules to Bone. J. Bone Miner Res..

[45] Moyano AL (2016). Sulfatides in extracellular vesicles isolated from plasma of multiple sclerosis patients. J. Neurosci. Res..

[46] Xu L (2013). Effects of cell surface α2-3 sialic acid on osteogenesis. Glycoconj J..

[47] Wilson KM, Thomas-Oates JE, Genever PG, Ungar D (2016). Glycan Profiling Shows Unvaried N-Glycomes in MSC Clones with Distinct Differentiation Potentials. Front. Cell Dev. Biol..

