# Supplementary material for: Exosome surface glycans reflect osteogenic differentiation of mesenchymal stem cells: Profiling by an evanescent field fluorescence-assisted lectin array system
Source: Sci Rep. 2019 Aug 8;9:11497. doi: 10.1038/s41598-019-47760-x (PMC6687741; doi:10.1038/s41598-019-47760-x)
Supplement: Supplementary file 1 — Supplementary Information [file 41598_2019_47760_MOESM1_ESM.docx]

**Supplementary Information Figures S1–5 and Supplementary Table 1**

**Exosome surface glycans reflect osteogenic differentiation of mesenchymal stem cells: Profiling by an evanescent field fluorescence-assisted lectin array system**

Asako Shimoda, Shin-ichi Sawada, Yoshihiro Sasaki, and Kazunari Akiyoshi^*^

Department of Polymer Chemistry, Graduate School of Engineering, Kyoto University, Katsura, Nishikyo-ku, Kyoto 615-8510, Japan

**Supplementary Figure S1. Full length western blot for Figure 3**

**
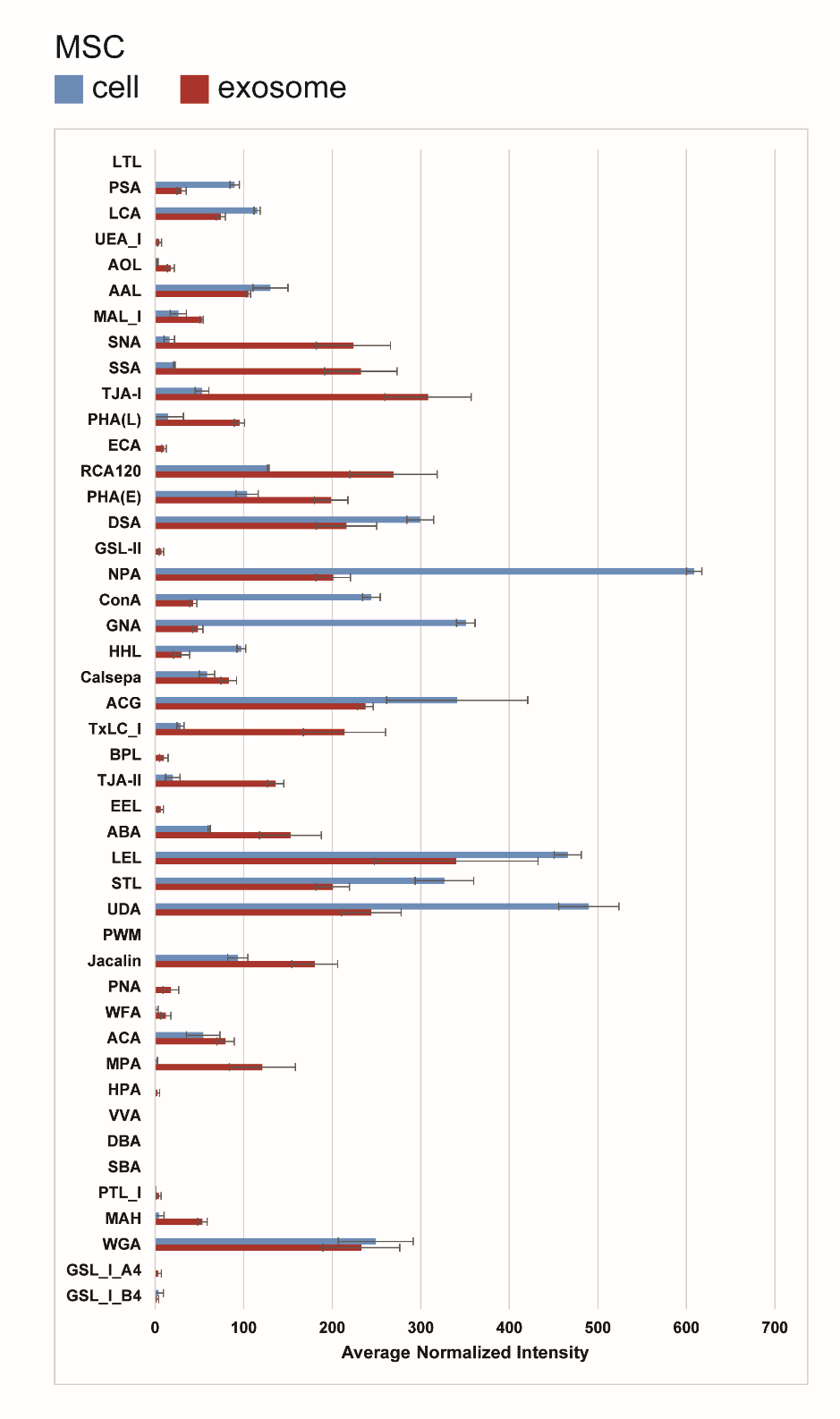
**

**Supplementary Figure S2. Glycan profile of exosomes and cell membranes from MSCs by an EFF-lectin array.**

**Each fluorescence intensity was normalized to the average intensities of all lectins. Data represent the mean ± SD (cells: n=2; exosomes: n=3).**

**
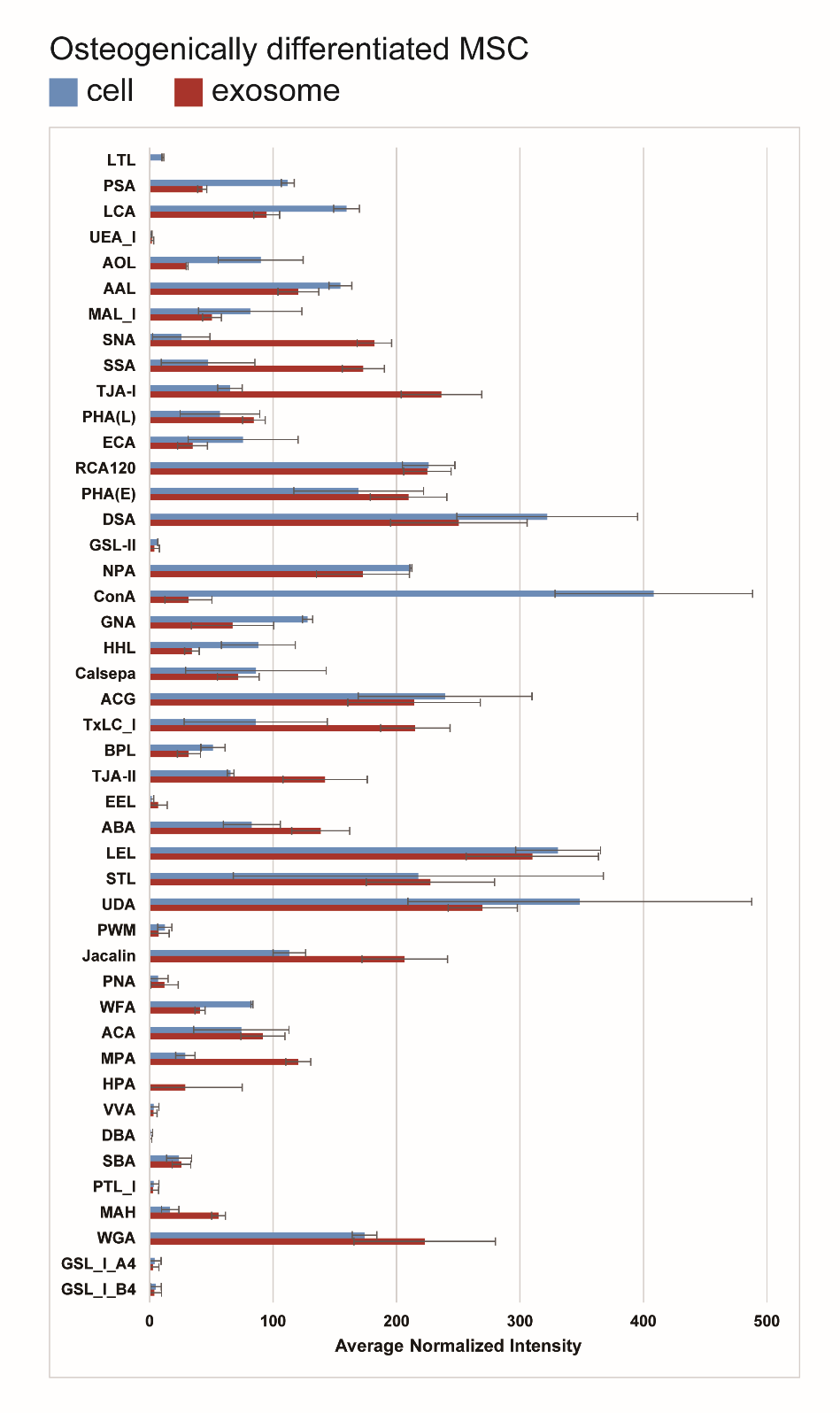
**

**Supplementary Figure S3. Glycan profile of exosomes and cell membranes from osteogenically differentiated MSCs on day 21.**

**Each fluorescence intensity was normalized to the average intensities of all lectins. Data represent the mean ± SD (cells: n=2; exosomes: n=3).**


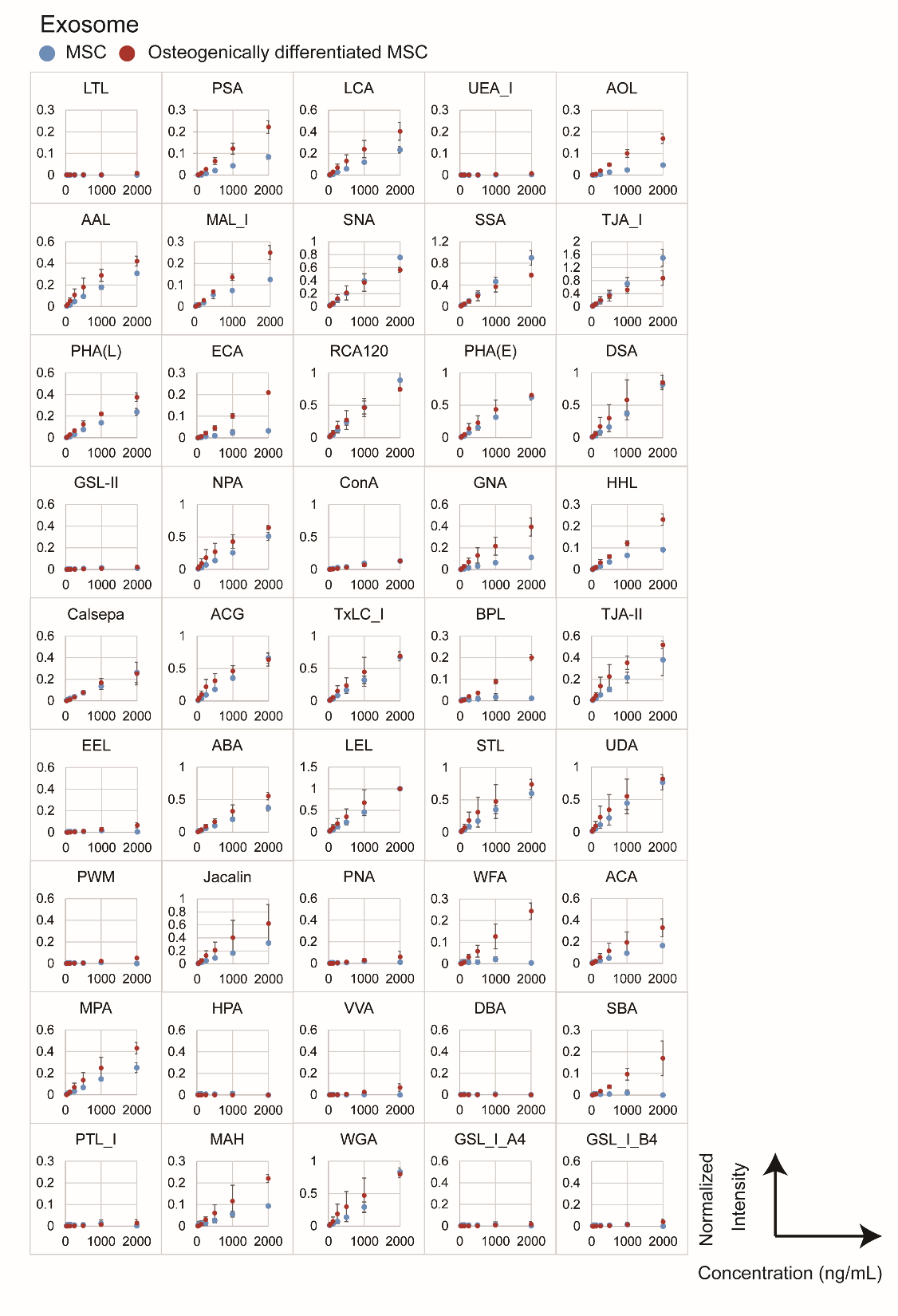


**Supplementary Figure S4. Binding curves of interactions between 45 lectins and exosomes. The resulting signals were normalized to the signal of LEL lectin. Data represent the mean ± SD of two independent experiments.**


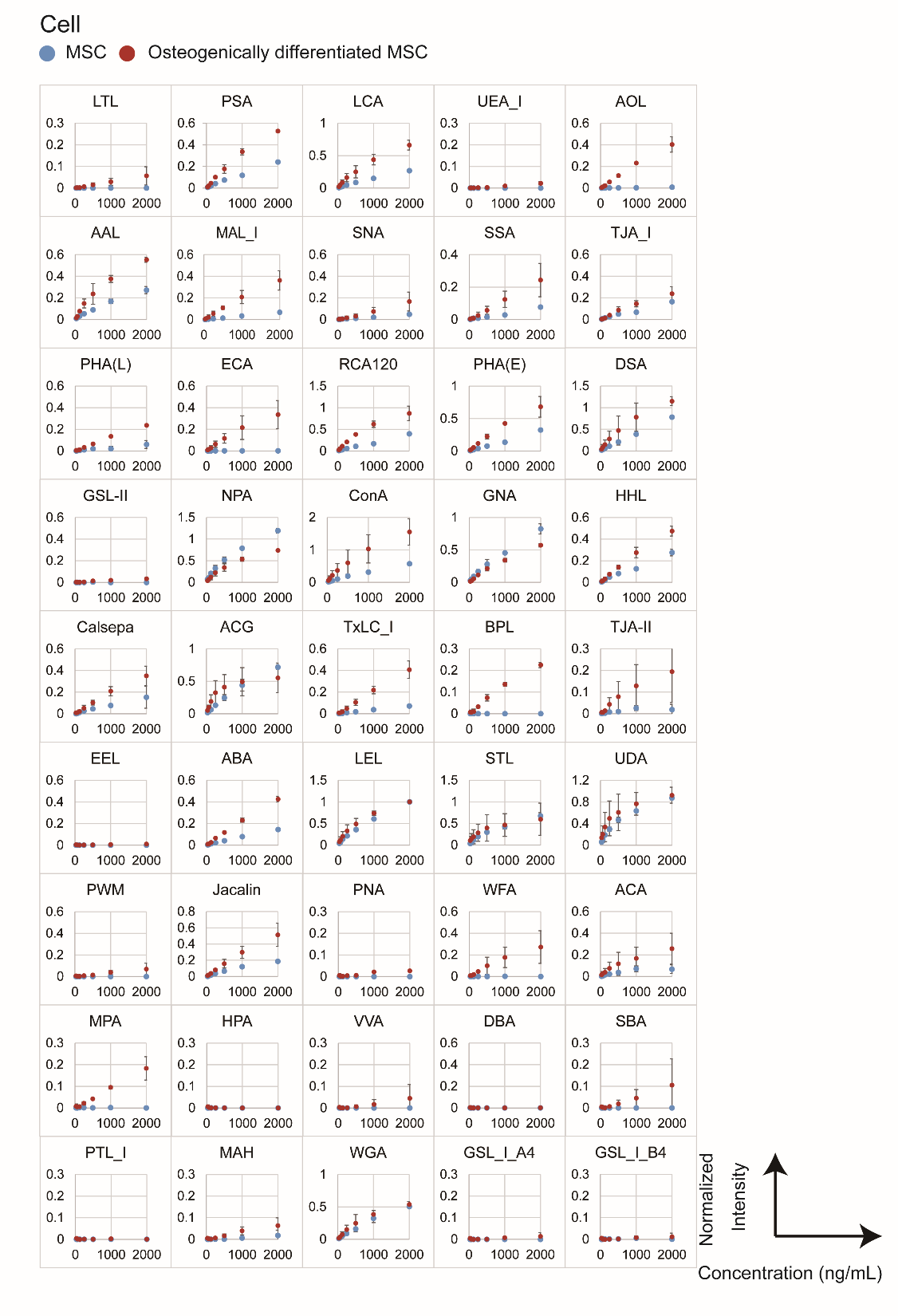


**Supplementary Figure S5. Binding curves of interactions between 45 lectins and cell membrane fractions. The resulting signals were normalized to the signal of LEL lectin. Data represent the mean ± SD of two independent experiments.**

| Supplementary Table 1. A list of lectins of LecChip^TM^ and glycan specificity. | | |
| --- | --- | --- |
| Lectin | *origin* | Glycan specificity |
| LTL | *Lotus tetragonolobus* | Fuc α1-3 GlcNAc |
| PSA | *Pisum sativum* | Core Fuc [Fuc α1-6] |
| LCA | *Lens culinaris* | Core Fuc [Fuc α1-6] |
| UEA_I | *Ulex europaeus* | Fuc α1-2 (Galβ1-4)GlcNAc |
| AOL | *Aspergillus oryzae* | Fuc α1-6 GlcNAc, Fuc α1-2 Galβ1-4GlcNAc |
| AAL | *Aleuria aurantia* | Fuc α1-6 GlcNAc, Fuc α1-3(Galβ1-4)GlcNAc |
| MAL_I | *Maackia amurensis* | Siaα2-3Gal |
| SNA | *Sambucus nigra* | Siaα2-6Gal, Siaα2-6GalNAc |
| SSA | *Sambucus sieboldiana* | Siaα2-6Gal |
| TJA-I | *Trichosanthes japonica* | Siaα2-6, Galβ1-4GlcNAc |
| PHA(L) | *Phaseolus vulgaris* | tri/tetra-antennary complex-type N-glycan |
| ECA | *Erythrina cristagalli* | Galβ1-4GlcNAc |
| RCA120 | *Ricinus communis* | Galβ1-4GlcNAc |
| PHA(E) | *Phaseolus vulgaris* | Bisecting GlcNAc |
| DSA | *Datura stramonium* | Galactosylated tetraantenary N-type |
| GSL-II | *Griffonia simplicifolia* | agalactosylated tri/tetra antennary glycans and GlcNAc |
| NPA | *Narcissus pseudonarcissus* | non-substituted α1-6Man |
| ConA | *Canavalia ensiformis* | α-Man (inhibited by presence of bisecting GlcNAc) |
| GNA | *Galanthus nivalis* | non-substituted α1-6Man |
| HHL | *Hippeastrum hybrid* | non-substituted α1-6Man |
| Calsepa | *Calystegia sepium* | α‐Man, bianntenary with bisecting GlcNAc |
| ACG | *Agrocybe cylindracea* | Gal β1-3Gal, Siaα2-3Galβ1-4GlcNAc |
| TxLC_I | *Tulipa gesneriana* | Manα1-3(Manα1-6)Man, bi/tri-antennary complex-type N-glycan,  GalNAc |
| BPL | *Bauhinia purpurea* | Galβ1-3GalNAc |
| TJA-II | *Tanthes japonica* | β-GalNAc, Fuc α1-2 Gal |
| EEL | *Euonymus europaeus* | Galα1-3Galβ-1-4GlcNAc, Fucβ-1-2Galβ1-3GlcNAc |
| ABA | *Agaricus bisporus* | Galβ1-3GalNAc, GlcNAc |
| LEL | *Lycopersicon esculentum* | Poly-LacNAc, (GlcNAc)n |
| STL | *Solanum tuberosum* | Poly-LacNAc, (GlcNAc)n |
| UDA | *Urtica dioica* | Poly-LacNAc, (GlcNAc)n |
| PWM | *Phytolacca Americana* | Poly-LacNAc, (GlcNAc)n |
| Jacalin | *Artocarpus integrifolia* | GlcNAcβ1-3GalNAc (Core3), Siaα2-3Galβ1-3GalNAc (sialyl T),  Galβ1-3GalNAc (T-antigen), α-GalNAc (Tn-antigen) |
| PNA | *peanut, Arachis hypogaea* | Galβ1-3GalNAc |
| WFA | *Wisteria floribunda* | GalNAcβ1-4GlcNAc (LacdiNAc), Galβ1-3(-6)GalNAc |
| ACA | *Amaranthus caudatus* | Galβ1-3GalNAcα-Ser/Thr (T) |
| MPA | *Maclura pomifera* | Galβ1-3GalNAcα-Ser/Thr (T),GalNAcα-Ser/Thr (Tn) |
| HPA | *Helix pomatia* | GalNAc (Tn) |
| VVA | *Vicia villosa* | GalNAc, GalNAcα-Ser/Thr (Tn) |
| DBA | *Dolichos biflorus* | GalNAcα-Ser/Thr (Tn), GalNAcα1-3GalNAc |
| SBA | *soybean, Dolichos biflorus* | Terminal GalNAc |
| PTL_I | *Psophocarpus tetragonolobus* | α-GalNAc and Gal |
| MAH | *Maackia amurensis* | Siaα2-3Galβ1-3(Siaα2-6)GalNAc |
| WGA | *wheat germ, Triticum aestivum* | (GlcNAc)n, polySia |
| GSL_I_A4 | *Griffonia simplicifolia* | GalNAc |
| GSL_I_B4 | *Griffonia simplicifolia* | αGal |
